# Epithelial to mesenchymal transition as a biomarker in renal fibrosis: are we ready for the bedside?

**DOI:** 10.1186/1755-1536-4-11

**Published:** 2011-04-06

**Authors:** Pierre Galichon, Alexandre Hertig

**Affiliations:** 1 Institut national de la santé et de la recherche médicale (INSERM), UMR S702, 4 rue de la Chine, Paris, 75020, France; 2 Université Pierre et Marie Curie, Sorbonne Universités, 4 place Jussieu, Paris, 75005, France; 3 Urgences néphrologiques et transplantation rénale de l'hôpital Tenon, assistance publique des hôpitaux de Paris, 4 rue de la Chine, Paris, 75020, France

## Abstract

Over the past two decades, the concept of the epithelial to mesenchymal transition (EMT) has been imported from embryology and oncology to fibrosis, particularly in the kidney. This interest in EMT in the context of renal fibrosis stems from observations of epithelial cells undergoing phenotypic changes reminiscent of fibroblasts. Whether EMT is actually a source of interstitial fibroblasts has been the subject of heated debate, and this controversy has caused physicians to neglect the value of EMT as a biomarker in renal fibrosis. In this review, we describe the evolution of the techniques used to detect EMT during fibrosing renal diseases, and what information they provide in the diagnosis of various renal diseases. Highlighting the great heterogeneity of these techniques and the need to standardize them, we warn against some misleading uses of EMT markers. We suggest using the association of vimentin and β-catenin for the diagnosis of EMT in renal pathology because it is both sensitive and prognostic, thus satisfying the properties required for a screening test. Finally, we discuss the potential interests to diagnose EMT for the comprehension of renal fibrosis and for clinical practice.

## Introduction: the concept of EMT

'What is simple is false, and what is complex is unusable'

Paul Valéry (1941)

Since its first description by Elisabeth Hay, epithelial to mesenchymal transition (EMT) has raised increasing interest. One reason for this is that the concept has extended from embryology to pathology. It was first defined in embryological studies as a process that is instrumental to organogenesis, in which cells lose their epithelial phenotype, acquire mesenchymal features, and migrate to generate new organs in the embryo [1]. This phenomenon, now called Type 1 EMT [2], is replicated in Type 3 EMT, conferring on cancerous cells the ability to disseminate by metastasis and to resist chemotherapy [3]. Type 2 EMT refers to the rather startling concept that epithelial cells subjected to injury may undergo similar transformations and thus provide new fibroblasts in the interstitium.

EMT was first associated with fibrogenesis 15 years ago, with the observation of renal tubular epithelial cells aberrantly expressing fibroblast-specific protein (FSP)1 in a model of mouse anti-tubular membrane disease[4]. This led Strutz *et al*. to hypothesize that some fibroblasts might be derived from transformed epithelial cells. This hypothesis was confirmed when Iwano showed that tubular epithelial cells bearing the reporter gene *lacZ *massively contributed to the pool of interstitial fibroblasts (up to 36% of all fibroblasts) in a model of mouse renal fibrosis induced by unilateral ureteral obstruction [5]. The study of renal EMT raised even more interest and hopes in the nephrological community 1 year later, when Zeisberg *et al*. showed that bone morphogenetic protein (BMP)7 could reverse EMT in mice exposed to nephrotoxic serum and even reverse renal fibrosis itself [6].

However, several studies have subsequently contested the reality of EMT in renal fibrosis. Fate-tracing experiments on tubular epithelial cells in various animal models (Habu venom plus angiotensin 2 in rats, unilateral ureteral obstruction or ischemia-reperfusion in mice) failed to identify a single fibroblast originating from the tubular epithelium [7,8]. At present, the existence of Type 2 EMT is debated so heatedly that its supporters and detractors seem irreconcilable [9].

In parallel, evidence has been sought for EMT in human renal diseases, mainly using immunohistochemistry. Obviously, studies in patients do not allow for definitive conclusions on all criteria defining EMT (especially the ability to migrate outside the basal membrane), which is why some authors, including us, have coined weaker terms such as 'partial EMT', 'EMT-like changes', 'epithelial phenotypic changes' or 'EMT-marker expression', thus avoiding the most controversial aspect of EMT. However, the evidence for EMT-marker expression during renal fibrosis has accumulated over time, and we believe that a consensus can now be reached on the presence of at least some features of EMT during tissue fibrosis, allowing us to translate part of the fundamental concept of EMT into clinical use as a biomarker.

### EMT: a misunderstood concept

A misinterpretation of epithelial plasticity can be traced to the belief that an epithelial cell undergoing EMT should become a new fibroblast. However, the definition of EMT does not require that an epithelial cell becomes any specific type of a cell. EMT is defined by phenotypic and functional changes that are reminiscent of mesenchymal cells. This in no way means that an epithelial cell will become a fibroblast. Strictly speaking, the term 'mesenchyme' is synonymous with 'primitive connective tissue', that is, any tissue that 1) is neither epithelial nor muscular nor nervous (see Ross *et al*, pages 54 and 115 [10]); and 2) the function of which is to provide an extracellular matrix (ECM) used as a support for these other cell types to function properly. As was recently emphasized, the migration of an epithelial cell out of the tubular structure into the interstitium where it will produce matrix (and thereby act as a new fibroblast) is but the 'extreme' form of EMT, not the essential criterion for the diagnosis of EMT [9,11].

### EMT reflects a global fibrogenesis

The term 'EMT' is by definition limited to the molecular events occurring in epithelial cells, but the presence of EMT reflects a more global process, affecting other cellular populations. Thus, the upregulation in the epithelium of the expression of mesenchymal genes is also seen in the renal interstitium. In IgA nephropathy, focal and segmental glomerulosclerosis or diabetic nephropathy, interstitial expression of α-smooth muscle actin (α-SMA) and FSP1 correlates with the progression of the disease, regardless of the origin of these FSP1-positive cells [12-14]. The reason why immunohistochemistry is of invaluable help and of high diagnostic value is that it is extremely sensitive in detecting the expression of mesenchymal proteins, specifically in highly differentiated epithelial cells subjected to some form of injury. The detection of structural markers is much easier when they are expressed *de novo *in cells, and more difficult when they are upregulated only in mesenchymal cells such as fibroblasts, which already have basal expression of such markers, in endothelial cells with a virtual cytoplasm, or in poorly defined cell types such as pericytes. Consistent with the idea that EMT is a part of a global fibrogenic process, the use of DNA microarrays has allowed quantitative measurement of the expression of mesenchymal genes in the total cortex from renal biopsies, and is 'generally' (that is, cortex-wide) upregulated in fibrosing allografts [15-17]. Similarly, preliminary results indicate that mRNA expression of EMT genes can be used as non-invasive surrogate markers for renal fibrosis in urine, even though the origin of the urinary cells that are being studied is not determined [18].

### FSP1: a historical but complex EMT marker

A milestone in the study of Type 2 EMT was the identification of FSP1 by Strutz *et al*., which has opened the door to the study of the epithelium as an active participant in tissue fibrosis[4]. Currently, the expression of FSP1 by the tubular epithelium is considered to be an incontrovertible criterion for the 'diagnosis' of EMT. We believe that this should be reconsidered for three reasons. First, the sensitivity of FSP1 as a fibroblast marker has been contested, as many authentic fibroblasts do not express it [14,19-21]. Second, its specificity is a persistent matter of debate, as several studies have reported that FSP1 can be expressed by leukocytes, particularly macrophages [19,21-26]. Even though the antibodies used to detect leukocytes in some of these studies have themselves overlapping specificities, a recent publication using a mouse model of liver injury showed that most FSP1-positive cells stained positively for F4/80, which is at present the most specific marker for macrophages [21]. Even though the aberrant expression of FSP1 by renal epithelial cells is still highly specific to fibrogenic renal diseases, this does not necessarily imply that FSP1-positive epithelial cells will generate a neofibroblast (incidentally, this is true for all EMT markers). Thus, although FSP1 has been shown to induce EMT *in vitro *[4,27,28], facilitating the metastasis of cancer cells, the consequences of its expression in the tubular epithelium during fibrogenesis remain to be determined [29]. Last, its detection by immunostaining within the renal epithelium undergoing EMT is rare [4,14,30] and sometimes technically difficult to obtain (for example, it will be negative if the fixative used is alcohol-formalin-acetic acid [30]). Overall, these observations disqualify FSP1 as a usable biomarker in kidney biopsies.

### Diagnosing EMT in tubular structures: practical considerations

It is important to use multiple markers simultaneously to detect the coordinated changes associated with EMT in the epithelial structures [31]. In humans, where only the observation of renal biopsies is possible, the migration of tubular cells outside of the basal membrane is obviously impossible to 'see'. Furthermore, the evidence that tubular cells display a decrease in the expression of epithelial markers is always questionable because the various tubule segments differentially express the classic epithelial proteins such as cadherins, cytokeratins and others, even under physiological conditions [32,33]. For example, E-cadherin is minimally expressed by proximal tubular cells in humans, thus the assertion that E-cadherin is lost or decreased necessarily implies that a distal tubule is being studied [30].

By contrast with animal studies the effective clinical diagnosis of EMT means that, rather than the loss of an epithelial phenotype, it is the aberrant acquisition of mesenchymal properties that is being detected in epithelial cells. Two kinds of EMT markers can be used in humans, and can be classified according to their structural (for example, changes in the composition of the cytoskeleton) or functional (, for example, ECM production) relevance. Schematically, the expression of structural proteins (for example, vimentin) is a feature present in quiescent mesenchymal cells, whereas the expression of functional proteins constitutes a feature of activated mesenchymal cells. The heterogeneity of these markers also has several advantages, as it allows the detection of EMT-like changes at different stages, depending on the characteristics of the disease and the sensitivity and specificity needed for diagnosis (Table 1). For example, vimentin is an intermediate-filament constituent of the cytoskeleton of mesenchymal cells. Its expression is associated with the phenotype of cells, specifically shape, motility and adhesion [34]. In tumors, it plays a role in apoptosis, invasion and metastasis, and is thus a new therapeutic target [35-37]. Vimentin knockout mice have delayed wound healing [38]. Vimentin seems to play a role in some, but not all, models of renal injury and repair [39,40]. It is not expressed in the normal adult tubular epithelium, but it is strongly expressed by by interstitial cells and the foot processes of podocytes, and faintly by endothelial cells [41,42]. This opportunely serves as an internal positive control. This marker lacks specificity as a mesenchymal marker, but it is very sensitive in that vimentin staining is absolutely negative in normal renal tubular cells and positive at a very early stage after injury. Importantly, expression of vimentin does not preclude the possibility of a reversible alteration of the epithelium; the epithelial expression of vimentin was seen in a model of transient renal injury followed by complete repair of tissue [43,44]. Regardless, the sensitivity of its detection makes it an excellent candidate for early screening of tubular injury.

**Table 1:** Various combinations of epithelial to mesenchymal transition (EMT) markers used in human studies

Study	**EMT markers used**^**1**^
Jinde [45]	α-SMA^1^+, cytokeratin+, collagens+

Rastaldi [46]	Vimentin+, α-SMA+, cytokeratin- ZO1-, P4H+, HSP47^3^+, collagen1+

Vongwiwatana [47]	E-cadherin-, cytokeratin-, vimentin+, S100A4+, α-SMA+, HSP47+

Vitalone [56]	Ecadherine+, α-SMA+ OR E-cadherin+, S100A4+

Hertig [30,52,57]	Vimentin+, translocation β-catenin+

^1^Nonexhaustive list of various combinations of markers used to detect EMT in humans. Note the contradictory criteria for EMT concerning epithelial markers.

^2^Smooth muscle actin.

^1^Heat-shock protein

Conversely, α-SMA, a marker of activated fibroblasts, and heat-shock protein (HSP)47, a marker of collagen production, are less sensitive markers, but are more clearly linked to fibrosis [45-47]. β-catenin is a marker of particular interest here; depending on its localization in the cell, it is either a structural epithelial marker or a mesenchymal functional marker. Thus, whereas β-catenin connects E-cadherin molecules to the actin cytoskeleton in epithelial cells that behave like epithelial cells, it may translocate in the cytosol and subsequently into the nucleus to act as a mesenchymal transcription factor in epithelial cells that engage in EMT. β-catenin was actually shown to be a key activator of EMT, notably within the Wnt pathway, in various experimental models such as ureteral obstruction, the Fisher to Lewis allograft model of renal transplantation, and adriamycin nephropathy [48-51]. Although any injury will probably trigger the expression of some structural proteins characteristic of fibroblasts, only an extended or repetitive injury will lead to a functional 'EMT' and thereby to the production of ECM by the surviving epithelium.

### EMT in human renal disease

The expression of EMT markers in native kidneys was investigated by Jinde on 127 patients with IgA nephropathy, rapidly progressive glomerulonephritis and minimal-change disease (MCD) [45]. One year later, another study evaluated 133 renal biopsies with nine different diagnoses [46]. Impressively, these two studies found frequent expression of EMT markers in all the renal diseases with the exception of MCD, where it was rare or absent. More than just a collateral phenomenon associated with renal disease, EMT markers were shown to be associated with the severity of interstitial fibrosis and the impairment of renal function [45,46]. In patients with multiple myeloma and the highly fibrogenic condition cast nephropathy, the expression of EMT markers was highest, including in morphologically preserved tubules [52].

By contrast, EMT-marker expression was not found in patients with overt proteinuria due to MCD [45,46,52], in keeping with the fact that the high proteinuria seen in MCD is rarely associated with interstitial fibrosis or renal failure [53]. This strongly supports the use of EMT as a biomarker of renal fibrogenesis. Furthermore, in the study by Rastaldi *et al*., EMT markers were also correlated with renal function in a subgroup of 45 patients with little interstitial fibrosis or infiltration, emphasizing the excellent sensitivity of these markers [46]. Other studies have focused on patients with one specific renal disease. In an ingenious study, Yamaguchi showed a correlation between EMT markers in urinary podocytes and the severity of diabetic nephropathy [54]. Ultimately, it is in renal allografts that the association of EMT markers with severity of renal disease has been best documented. Vongwiwatana showed that EMT markers were associated with serum creatinine, proteinuria and a history of T-cell-mediated rejection [47]. In another set of patients with stable renal function 3 months after transplantation, we found that EMT markers were associated with higher serum creatinine, cold-ischemia time and subclinical acute rejection [30].

## EMT markers have a prognostic value in renal allografts

**Figure 1:**
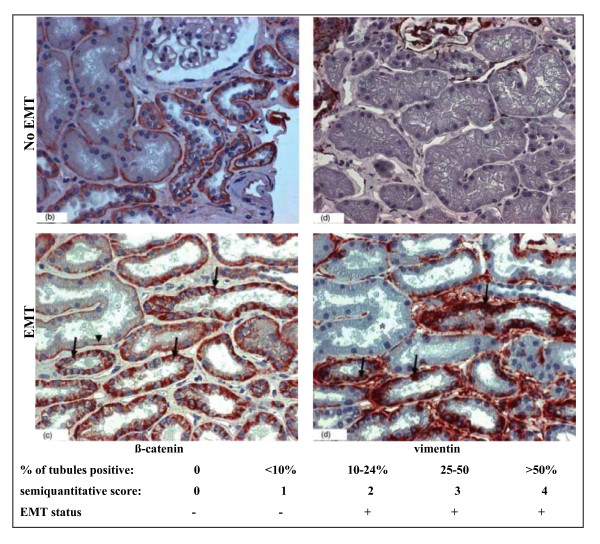
**Detection of epithelial to mesenchymal transition (EMT) by β-catenin and vimentin immunostaining, and definition of the subsequent prognostic EMT score**. Expression of the EMT markers β-catenin and vimentin in renal allograft biopsies at implantation (no EMT) and 3 months after implantation (EMT). Note that the normal basal linear expression of β-catenin became irregular and extended to the cytoplasm, and that vimentin was expressed *de novo *in the EMT-positive tubules.

By definition, sequential biopsies are needed to study the correlation of EMT-marker expression with fibrogenesis, that is, with the progression of fibrosis over time. These data are available in renal transplantation because kidney recipients are given iterative surveillance biopsies [55]. Two studies have evaluated the prognostic value of EMT-marker expression in renal allografts, but their conclusions were contradictory. The first study showed no correlation between the expression of EMT markers at 1 month after transplant and the presence of tubular-interstitial injury at 3 months [56]. Our interpretation is that EMT-marker expression at 1 month is related to acute and transient factors linked to ischemia-reperfusion injury or to the surgical transplantation procedure, with little relevance to the long-term outcome of the graft. Moreover, in this study, EMT was defined as staining for either FSP1 or α-SMA in a tubular cell simultaneously stained for E-cadherin. This diagnostic criterion is questionable, because 1) E-cadherin expression is classically downregulated during EMT and absent in cells after complete EMT; and 2) as previously noted, the sensitivity of α-SMA and FSP1 as EMT markers is poor, because their late and/or rare expression during human EMT. This choice of markers thus probably selected for a minor population of cells. In another study, we found that the expression of two other EMT markers mentioned above, vimentin and β-catenin, 3 months after transplantation, do have prognostic value, and were associated with a more rapid progression towards graft interstitial fibrosis and decrease in renal function at 12 months. In addition, EMT-marker expression and interstitial or tubular inflammation were the only factors associated with fibrosis at 12 months in a multivariate analysis [57]. These results were confirmed by a recent study in which vimentin expression within the tubular epithelium was associated with more chronic interstitial fibrosis and tubular atrophy and with poorer graft function, with a median follow-up of 5 years [58]. We believe that the choice of β-catenin and vimentin is judicious, because they are expressed early in the course of tubular injury [43,44] and the immunohistochemical techniques are robust (Figure 1).

## Perspectives

Although EMT is a pathological process common to many fibrosing nephropathies, the question remains as to whether there are one or several EMTs. Currently, it is not known if some EMT markers have a higher specificity for a specific renal disease, as studies comparing the expression of EMT markers between various human pathologies are few. Mechanistically, however, EMT can be triggered by various master molecules that are each sufficient to induce EMT [59,60]. For example, glycogen synthase kinase 3β is a kinase that serves as a common effector of multiple EMT pathways, facilitating the efficiency of Snail and β-catenin as pro-EMT transcription factors [61]. Hypoxia can cause EMT by inducing snail, but snail is not specific to hypoxia, and other EMT master genes can be induced separately by hypoxia [62-64]. Although identification of a unique 'master switch' [60] would be an interesting target for the treatment of renal fibrosis, multiple and disease-specific molecules could be preferable as biomarkers.

Anti-EMT strategies are emerging as potential anti-fibrotic treatments. As the two processes are closely linked, it is difficult to determine whether a treatment is anti-EMT or anti-fibrotic. Thus, TGF-β is known both as an EMT inducer and as a pro-fibrotic molecule, and in their spectacular study using BMP7 to antagonize EMT, Zeisberg *et al*. showed that both tubular EMT and fibrosis could be reversed, but they pointed out that this association does not allow the conclusion that fibrosis was reversed through an anti-EMT effect [6]. Nonetheless, this was probably the most promising experimental therapy in the field for decades, and showed that EMT markers were useful to monitor the epithelial damage *in vivo*. Other potential anti-fibrosis treatments have been tested for their anti-EMT effect (for example, hepatocyte growth factor [65], paricalcitol [49] and juglone [66-68].

## Conclusions

There is sufficient evidence to assert that at least some EMT markers are expressed during renal fibrosis. Such biomarkers are very useful compared with conventional histology, because they detect fibrogenesis as opposed to fibrosis. They are very sensitive, and are expressed at an early stage of the disease. This sensitivity is particularly obvious for vimentin expression, which is currently being translated into clinical practice in a study (clinical trial NCT#01079143) using vimentin and β-catenin tubular staining for the screening of early tubular injury in kidney-graft biopsies. Such early markers are also useful for renal research, as they allow characterization and study of various models of renal injury at a stage closer to the initiating mechanism of the disease. Second, they are markers with reasonable prognostic value. Were an anti-fibrotic molecule to be introduced into the pharmacopeia, EMT markers would help in identification of high-risk patients, and also provide a short-term endpoint criterion to evaluate the efficacy of therapeutic strategies. Finally, the concept of EMT has positively stimulated research on adult-tissue fibrogenesis, and helped to characterize the pro-fibrotic molecular environment.

## Competing interests

The authors declare that they have no competing interests.

## Authors' contributions

Both authors participated in the conception and approved the final manuscript. PG drafted the manuscript. AH revised the manuscript.
